# The antiviral restriction factor IFN-induced transmembrane protein 3 prevents cytokine-driven CMV pathogenesis

**DOI:** 10.1172/JCI84889

**Published:** 2017-02-27

**Authors:** Maria A. Stacey, Simon Clare, Mathew Clement, Morgan Marsden, Juneid Abdul-Karim, Leanne Kane, Katherine Harcourt, Cordelia Brandt, Ceri A. Fielding, Sarah E. Smith, Rachael S. Wash, Silvia Gimeno Brias, Gabrielle Stack, George Notley, Emma L. Cambridge, Christopher Isherwood, Anneliese O. Speak, Zoë Johnson, Walter Ferlin, Simon A. Jones, Paul Kellam, Ian R. Humphreys

**Affiliations:** 1 Institute of Infection and Immunity, School of Medicine, Cardiff University, Heath Park, Cardiff, United Kingdom.; 2 Wellcome Trust Sanger Institute, Hinxton, Cambridgeshire, United Kingdom.; 3 Novimmune SA, Geneva, Switzerland.

## Abstract

The antiviral restriction factor IFN-induced transmembrane protein 3 (IFITM3) inhibits cell entry of a number of viruses, and genetic diversity within *IFITM3* determines susceptibility to viral disease in humans. Here, we used the murine CMV (MCMV) model of infection to determine that IFITM3 limits herpesvirus-associated pathogenesis without directly preventing virus replication. Instead, IFITM3 promoted antiviral cellular immunity through the restriction of virus-induced lymphopenia, apoptosis-independent NK cell death, and loss of T cells. Viral disease in *Ifitm3^–/–^* mice was accompanied by elevated production of cytokines, most notably IL-6. IFITM3 inhibited IL-6 production by myeloid cells in response to replicating and nonreplicating virus as well as following stimulation with the TLR ligands Poly(I:C) and CpG. Although IL-6 promoted virus-specific T cell responses, uncontrolled IL-6 expression in *Ifitm3^–/–^* mice triggered the loss of NK cells and subsequently impaired control of MCMV replication. Thus, IFITM3 represents a checkpoint regulator of antiviral immunity that controls cytokine production to restrict viral pathogenesis. These data suggest the utility of cytokine-targeting strategies in the treatment of virus-infected individuals with impaired IFITM3 activity.

## Introduction

Antiviral immune responses elicited following acute viral infections are tightly regulated to limit uncontrolled immune pathology, while ensuring adequate control of the primary infection. Herpesvirus infections are typically controlled by asymptomatic resolution of the primary infection and establishment of viral latency, whereby the adaptive immune response controls replication of reactivating virus. Thus, host innate and adaptive immune mechanisms work to hold herpesvirus replication in check. In some settings, a failure in antiviral defense during the primary infection leads to elevated viral replication and virus-induced disease pathology (1–3).

Herpesvirus restriction of immune activation may contribute to limited pathology during acute infection. Indeed, a clear evolutionary advantage for herpesviruses exists to modulate antiviral immunity to maintain host fitness during acute infection, but also to facilitate persistence and the establishment of latency. The β-herpesvirus human CMV (HCMV) represents a paradigm for viral immune evasion. It encodes numerous proteins with putative immune-modulatory actions (4, 5), and HCMV infection profoundly influences the expression of host immune–related proteins (6). Studies using the murine CMV (MCMV) model of β-herpesvirus infection have highlighted that CMV also exploits host immune–inhibitory mechanisms, including the immune-regulatory cytokine IL-10, to facilitate viral persistence (7–9). Paradoxically, both cellular (10) and viral (11) IL-10 restrict acute pathologies in experimental models of CMV infection. Consequently, a delicate and important balance exists among the control of acute replication, virus-induced inflammation, and viral persistence. The factors governing this balance and the potential influence that the host and virus genetic variation exerts on this process require a better understanding.

IFN-induced transmembrane protein 3 (IFITM3) is an IFN-inducible antiviral restriction factor that is enriched in endosomal compartments (12). IFITM3 restricts endocytosis-dependent entry of diverse viruses, most notably influenza, dengue virus, West Nile virus, and HIV (13, 14). Importantly, genetic studies emphasize the pivotal role that IFITM3 plays in governing viral disease in humans. A number of polymorphisms within human IFITM3 have been identified that may potentially influence IFITM3 function (15). Notably, the minor rs12252-C allele in IFITM3, which has an allelic frequency of 0.03 and 0.5 in European white and Han Chinese populations, respectively (16, 17), is associated with impaired restriction of influenza replication (15, 16, 18), increased susceptibility to severe influenza-associated disease (16, 17), and HIV progression (19).

Studies using murine infection models have highlighted a critical role for IFITM3 in restricting viral pathogenesis in vivo. *Ifitm3^–/–^* mice exhibit increased susceptibility to infection with influenza (16, 20), arthritogenic and encephalitic alphaviruses (21), respiratory syncytial virus (22), and West Nile virus (23). Susceptibility is associated with the significant impairment of the direct control of viral replication, in accordance with the established role for IFITM3 as an antiviral restriction factor. Interestingly, however, alterations in immune responses have also been described in these models (16, 21–23). While these observations suggest a possible link between IFITM3 and the regulation of antiviral immunity, the direct impact of IFITM3 on viral replication has not been disentangled from any immune-regulatory functions of IFITM3. Furthermore, studies of influenza infection have revealed that impaired antiviral immune responses in *Ifitm3^–/–^* mice can occur as a consequence of unregulated infection of immune cells (24, 25).

IFITM3 does not directly impinge on HCMV replication in vitro (26, 27). Consequently, we sought to establish whether IFITM3 influences herpesvirus pathogenesis in vivo. Using the MCMV model of infection, we determined that murine IFITM3 is a critical checkpoint regulator of herpesvirus-induced immune pathology during acute and chronic infection in vivo. Consistent with observations in HCMV infection, IFITM3 did not directly restrict MCMV replication, but instead acted to limit the overexuberant production of cytokines, in particular IL-6. Thus, IFITM3 activity acts as a rheostat of antiviral immunity that determines the pathological outcome of acute MCMV infection.

## Results

### IFITM3 determines the primary outcome of MCMV infection.

**Figure 1:**
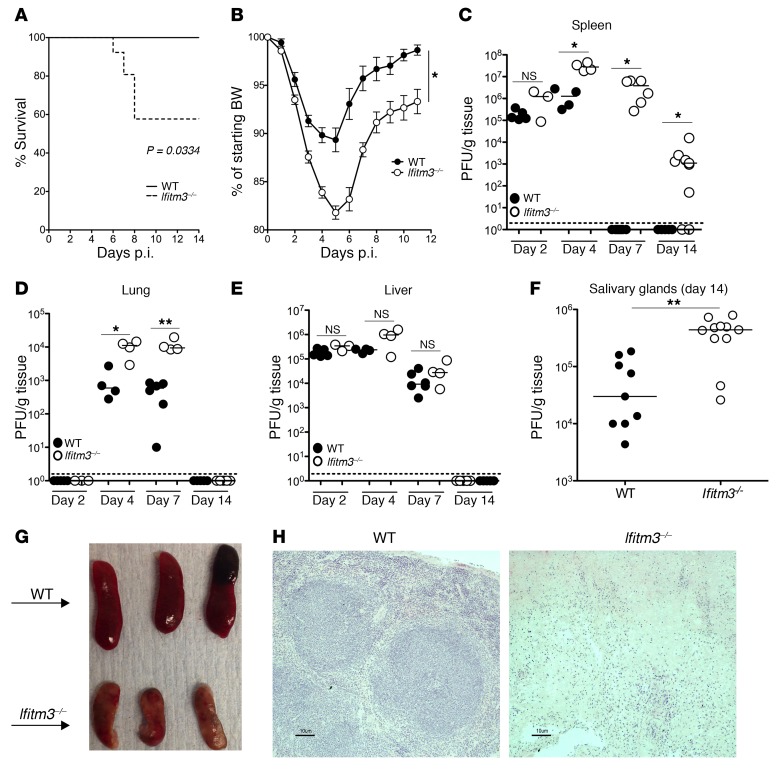
IFITM3 affords critical protection from MCMV infection. WT and *Ifitm3^–/–^* mice were infected with 3 × 10^4^ PFU MCMV, and survival (**A**) and weight loss (**B**) were assessed over time. Survival data include mice culled according to UK Home Office restrictions on virus-induced weight loss. Data shown are from 14 (WT) and 21 *(Ifitm3^–/–^*) mice per group merged from 3 experiments. *P* value in (**A**) determined by Mantel Cox test. Replicating virus in spleen (**C**), lung (**D**), liver (**E**) and salivary glands (**F**) on day 4 (**C**–**E**) or 14 (**F**) p.i. was quantified by plaque assay. (**G**) Spleen morphology in WT and *Ifitm3^–/–^* mice 14 days p.i. (**H**) Spleens were taken 14 days p.i. and sections stained with H&E. Original magnification, ×20; scale bars: 10 μm. Data are representative of at least 3 separate experiments. *P < 0.05 and **P < 0.01, by 1-way ANOVA (**B**) and by Mann Whitney-U (**C**–**F**). Error bars indicate ± SEM.

To assess the impact of IFITM3 on herpesvirus pathogenesis, we first infected control WT and *Ifitm3*-deficient mice with MCMV (using Salivary gland propagated Smith Strain MCMV [ATCC]). Infection of WT mice with 3 × 10^4^ PFU salivary gland–propagated Smith strain MCMV resulted in a nonlethal infection (Figure 1A) and moderate (~10%) weight loss (Figure 1B). However, *Ifitm3^–/–^* mice had only a 60% survival rate, with mice succumbing to infection or being culled, in accordance with UK Home Office guidelines regarding disease severity and weight loss, between post-infection (p.i.) days 6 and 8 (Figure 1, A and B). Surviving *Ifitm3^–/–^* mice also had delayed weight loss recovery that was still evident 12 days p.i. (Figure 1B). Furthermore, subclinical infection with an inoculum of 5 × 10^3^ PFU per mouse also induced weight loss in *Ifitm3^–/–^* mice but not in WT mice (Supplemental Figure 1A; supplemental material available online with this article; https://doi.org/10.1172/JCI84889DS1).

Exacerbated MCMV-induced weight loss in *Ifitm3^–/–^* mice was accompanied by a statistically significant higher viral load from day 4 p.i. in the spleen (Figure 1C) and lungs (Figure 1D), but not the liver (Figure 1E), after infection with standard inoculum (3 × 10^4^ PFU). Weight loss in *Ifitm3^–/–^* mice induced following low-dose inoculum (5 × 10^3^ PFU) also resulted in a significantly increased viral load in the spleen 4 days p.i. (Supplemental Figure 1B). Furthermore, 14 days after infection with standard inoculum, viral persistence in the salivary gland was evident in WT and *Ifitm3^–/–^* mice, but *Ifitm3^–/–^* mice harbored an elevated viral load in this established site of persistent MCMV replication (Figure 1F). Moreover, extensive pathology in the spleens of *Ifitm3^–/–^* mice was observed 14 days p.i. (Figure 1G), with evidence of severe disruption of follicular structures (Figure 1H). Spleens were not recoverable from *Ifitm3^–/–^* mice 3 months p.i. (data not shown), demonstrating the irreversible nature of organ damage. Thus, IFITM3 promoted host survival and control of viral replication during CMV infection.

### IFITM3 does not influence MCMV cell entry or infectious virion production.

**Figure 2:**
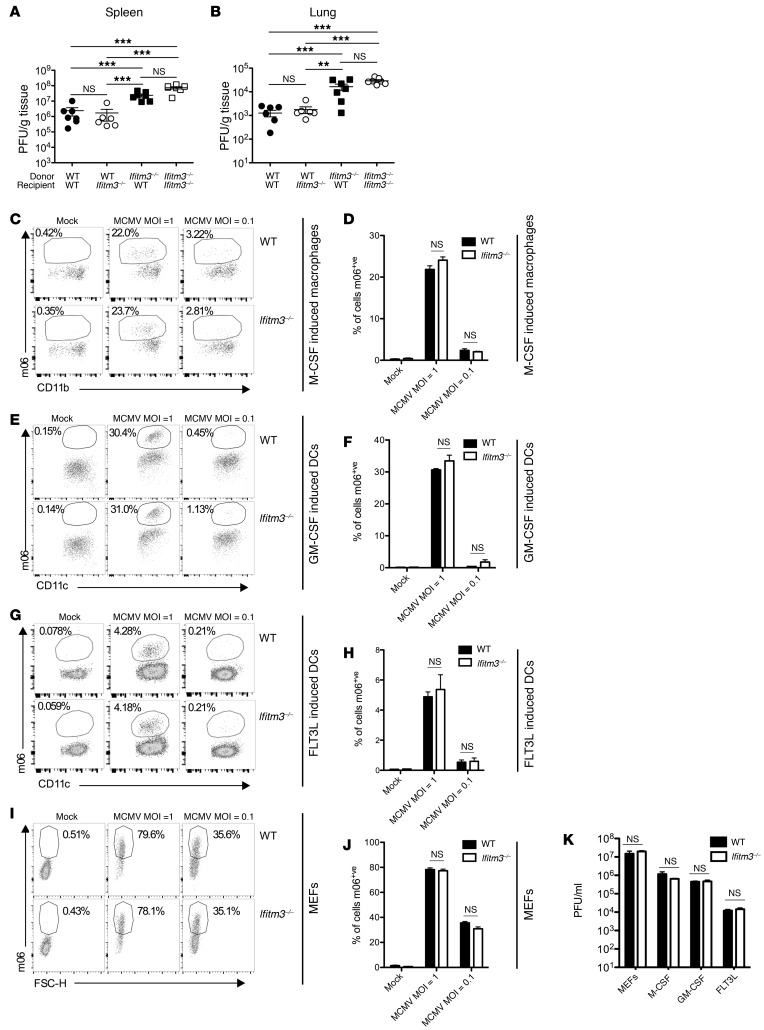
IFITM3 does not restrict MCMV replication. Mixed WT/*Ifitm3*^–/–^ bone marrow chimeras were generated and infected with MCMV, and after 4 days, PFU in spleen (**A**) and lung (**B**) were measured. Individual mice ± the median are shown from 1 of 2 experiments. M-CSF– (**C** and **D**), GM-CSF– (**E** and **F**), and FLT3L-differentiated myeloid cells (**G** and **H**) derived from WT and *Ifitm3^–/–^* bone marrow and WT and *Ifitm3^–/–^* MEFs (**I** and **J**) were infected with MCMV (using the pSM3fr-MCK-2fl BACmid) at different MOI, and MCMV m06 protein was detected 24 hours later by flow cytometry. FSC-H, forward scatter height. Data represent 2–3 experiments, and **B**, **D**, **F**, and **H** show the mean ± SEM of quadruplet wells. (**K**) WT and *Ifitm3*^–/–^ MEFs, M-CSF–, GM-CSF–, and FLT3L-differentiated myeloid cells were infected with pSM3fr-MCK-2fl MCMV at an MOI of 1. Supernatant was taken 4 days later, and replicating virus was quantified by plaque assay. Results are representative of 2 to 4 experiments. **P < 0.01 and ***P < 0.001, by 1-way ANOVA with Bonferonni’s multiple comparison post-test analysis (**A** and **B**) and by 2-tailed Students *t* test (**D**, **F**, **H**, **J**, **K**). m06^+ve^; positive intracellular staining for MCMV m06 protein.

IFITM3 restricts entry of a number of RNA viruses that utilize the endocytic pathway during cell entry (13). However, IFITM3 does not restrict HCMV entry into fibroblasts or epithelial cells (26, 27), the latter of which require endocytosis (28). Bone marrow chimera experiments revealed that IFITM3 deficiency within the hematopoietic cell compartment was sufficient to increase viral load 4 days p.i. in vivo (Figure 2, A and B). CMV does not productively infect lymphocytes (29), and we detected no MCMV infection of WT or *Ifitm3^–/–^* T cells or NK cells, as evidenced by the absence of MCMV m06 early protein in vitro (data not shown). Interestingly, MCMV infection of macrophage-CSF–differentiated (M-CSF–differentiated), granulocyte-macrophage CSF–differentiated (GM-CSF–differentiated), and FMS-like tyrosine kinase 3 ligand–differentiated (FLT3L-differentiated) bone marrow–derived myeloid cells was prevented by pretreatment with the endocytosis inhibitor 5-(*N*-ethyl-*N*-isopropyl)amiloride (EIPA) (Supplemental Figure 1, C–E). Thus, we investigated whether IFITM3 influences endocytosis-dependent MCMV replication within myeloid cells. MCMV entry was unaffected by IFITM3 deficiency when cells were infected at an MOI that resulted in nonsaturating infection in WT cells (MOI of 0.1 or 1, Figure 2, C–H) or following infection with a higher MOI (MOI = 10, Supplemental Figure 1F). Furthermore, IFITM3 had no impact on type I IFN–mediated endocytosis-dependent control of MCMV infection (Supplemental Figure 1, G–I). Moreover, MCMV infection of primary mouse embryonic fibroblasts (MEFs) was also unaffected by the absence of IFITM3 (Figure 2, I and J). Thus, in contrast to influenza infection (Supplemental Figure 1J), IFITM3 did not influence the infection efficiency of MCMV in our assays. Furthermore, we found that productive viral replication following infection of all cell types examined was also unaffected by IFITM3 deficiency (Figure 2K). Thus, as observed in HCMV infection (26, 27), our data suggest that IFITM3 does not directly restrict MCMV cell entry or subsequent replication.

**Figure 3:**
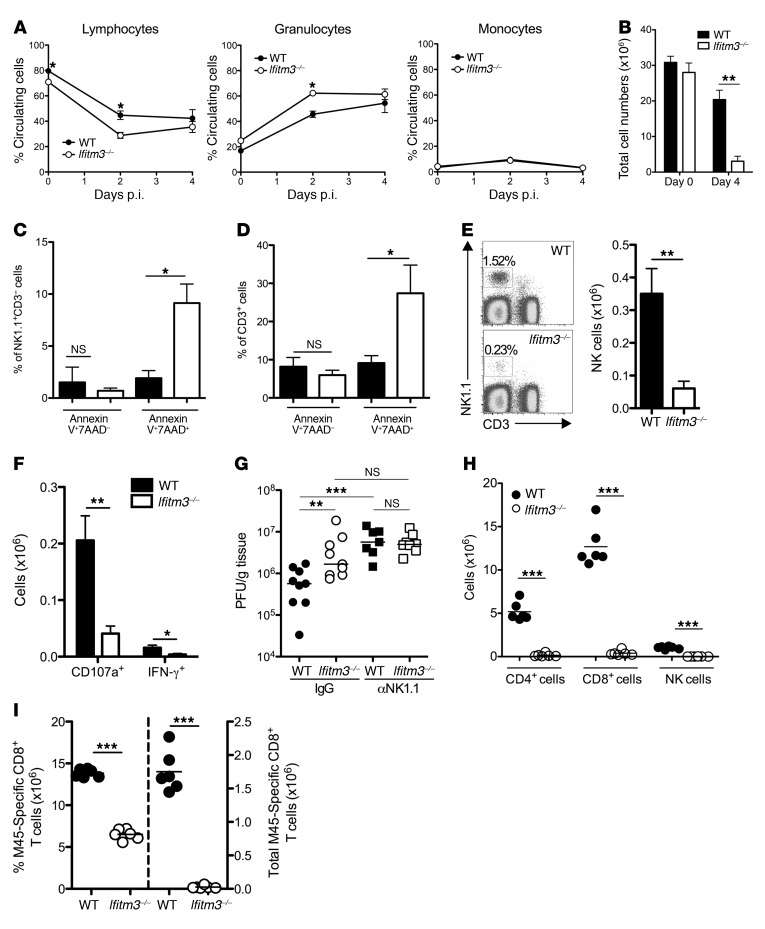
IFITM3 deficiency leads to impairment of cellular immunity. WT and *Ifitm3*^–/–^ mice were infected with MCMV. (**A**) On days 0, 2, and 4, the frequencies of circulating leukocyte populations in blood were quantified. Data represent the mean ± SEM of 3 mice per group for 3 experiments. (**B**) Viable splenocytes were quantified on day 4 p.i. Data represent the mean ± SEM of 3 to 9 mice per group for at least 5 experiments. NK1.1^+^CD3^–^ cells (**C**) and CD3^+^ cells (**D**) were stained with 7AAD and annexin V. Data represent the mean ± SEM of 4 to 6 mice per group for at least 3 experiments. (**E**) Representative bivariate flow plots of NK1.1 versus CD3, gated on viable cells (left), and total viable NK cells (right) on day 4 p.i. Data represent the mean ± SEM of 3 to 9 mice per group for at least 5 experiments. (**F**) The total number of NK cells positive for CD107a or intracellular IFN-γ was quantified by flow cytometry on day 4 p.i. Data represent the mean ± SEM of 8 to 9 mice per group for 3 experiments. (**G**) WT and *Ifitm3^–/–^* mice were depleted of NK cells, and the splenic viral load was assessed by plaque assay 4 days later. Data represent individual mice ± the median for 3 experiments (2 using anti-NK1.1 [αNK1.1] and 1 using anti-ASGM1 treatment). (**H**) The number of CD4^+^, CD8^+^, and NK1.1^+^ cells was quantified in spleens on day 7 p.i. Data represent individual mice ± the mean for 3 similar experiments. (**I**) Percentage and total M45-specific CD8^+^ T cells 7 days p.i.. All results represent at least 3 experiments. *P < 0.05, **P < 0.01, and ***P < 0.001, by 2-tailed Students *t* test (**A**–**D**, **F**, **H**, **I**) and by 1-way ANOVA with Bonferonni’s multiple comparison post-test analysis (**G**).

*Ifitm3^–/–^mice exhibit exacerbated lymphopenia and increased leukocyte death during MCMV infection*. We investigated the mechanisms underpinning MCMV-induced pathogenesis in *Ifitm3^–/–^* mice. Lymphopenia is a hallmark of severe viral diseases (30). We found that MCMV-infected *Ifitm3^–/–^* mice had exacerbated systemic lymphopenia and a concomitant elevation of circulating granulocytes during MCMV infection (Figure 3A). This was accompanied by a large reduction in splenocyte numbers 4 days p.i. (Figure 3B). Circulating blood platelets and red blood cells were not reduced in *Ifitm3^–/–^* mice, suggesting that lymphopenia was not a consequence of generalized bone marrow suppression (Supplemental Figure 2, A and B). Furthermore, concentrations of chemokines including the lymphocyte-attracting chemokines CCL5 and CXCL10 within the spleens of *Ifitm3^–/–^* mice were elevated as compared with concentrations detected in WT controls (Supplemental Figure 2C), indicating that tissue lymphopenia was probably not due to impaired chemokine-mediated cellular recruitment. Instead, cell death analysis revealed enrichment of late apoptotic/necrotic (annexin V^+^7AAD^+^) NK cells (Figure 3C) and CD3^+^ cells (Figure 3D) on day 4 p.i. in *Ifitm3^–/–^* mice. In contrast, we observed no significant enrichment of early apoptotic NK1.1^+^ or CD3^+^ cells, as detected by either caspase 3 expression or annexin V^+^7AAD^–^ staining either 2 or 4 days p.i. (M. Stacey and I. Humphreys, unpublished observations). Collectively, these data implied that apoptosis-independent cell death was a significant factor underpinning lymphopenia in MCMV-infected *Ifitm3^–/–^* mice.

### Impaired cellular immune responses in MCMV-infected Ifitm3^–/–^ mice.

We examined the influence of cellular immunity on the outcome of MCMV infection in *Ifitm3^–/–^* mice. Neutrophil depletion exacerbated MCMV-induced weight loss in both WT and *Ifitm3^–/–^* mice (Supplemental Figure 2D), consistent with their antiviral function (31) and the conclusion that, as suggested by elevated granulocyte responses in *Ifitm3^–/–^* mice (Figure 3A), impaired neutrophil responses were not responsible for the increased susceptibility of *Ifitm3^–/–^* mice to viral pathogenesis.

NK cells afford critical protection from HCMV (32) and MCMV (2) infections. Increased MCMV replication in *Ifitm3^–/–^* mice was accompanied by a 5-fold reduction in NK cell numbers (Figure 3E), which was similar to the reduction in splenocytes observed in *Ifitm3^–/–^* mice (Figure 3B). This equated to a comparable defect in the accumulation of degranulating (CD107a^+^) and IFN-γ^+^ (Figure 3F) NK cells, although frequencies of NK cells spontaneously expressing IFN-γ^+^ were low in both groups, which is consistent with the dominant role of perforin-mediated control of MCMV replication in the spleen (33). This finding is consistent with the conclusion that IFITM3 deficiency impinged on antiviral NK cell responses by influencing NK cell accumulation and survival rather than by directly influencing NK cell function. Notably, NK cell depletion using established methodology (34) resulted in a comparable virus load in WT and *Ifitm3^–/–^* mice 4 days p.i. (Figure 3G), suggesting that the NK cell defect significantly contributed to acute MCMV replication and pathology in *Ifitm3^–/–^* mice. In accordance, *Ifitm3^–/–^* mice did not show impaired control of a strain of MCMV lacking the m157 protein (Supplemental Figure 2E) that specifically induces NK cell activation (35).

During the latter stages of MCMV infection (day 7 p.i.), in accordance with substantial CD3^+^ cell death at early time points (Figure 3D), CD4^+^ and CD8^+^ T cell (Figure 3H) and virus-specific CD8^+^ T cell (Figure 3I) numbers were also drastically reduced in *Ifitm3^–/–^* mice, as were NK cell numbers (Figure 3H). Therefore, the broad protection afforded by IFITM3 for T cell survival in vivo promoted the generation of virus-specific T cell immunity.

### IFITM3 regulates MCMV-induced cytokine production.

**Figure 4:**
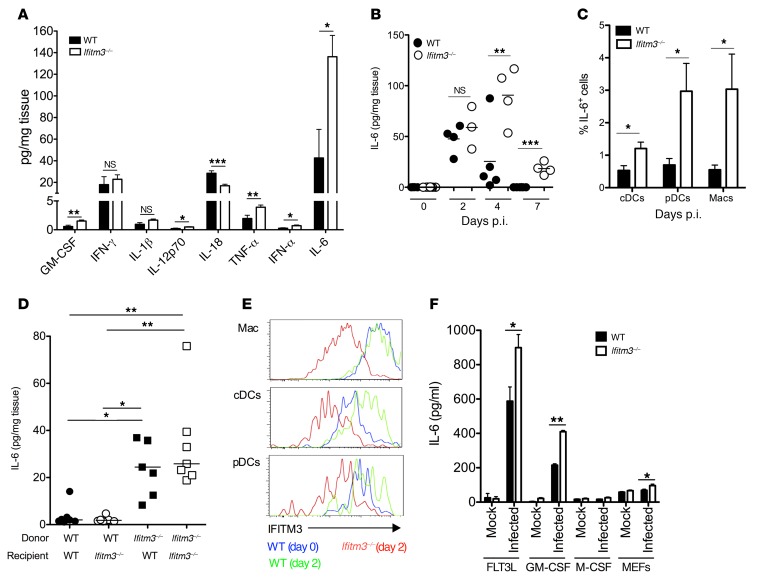
IFITM3 suppresses MCMV-induced IL-6 production. (**A**–**C**) WT and *Ifitm3^–/–^* mice were infected or not with MCMV. (**A**) Proinflammatory cytokines were measured by multiplex immunoassay in splenic homogenates 4 days p.i. Data represent the mean ± SEM of 8 to 9 mice per group. (**B**) Spleens were taken on days 0, 2, and 4 p.i., and IL-6 was measured by ELISA in tissue homogenates. Individual mice ± mean values are depicted, and the data represent at least 2 experiments for each time point. (**C**) IL-6 expression by (unstimulated ex vivo) CD11c^h^iMHC II^+^ (cDCs), CD11b^–^CD11c^+^B220^+^Siglec H^+^ (pDCs), and F4/80^+^CD11b^+^ (Macs) was detected by flow cytometry. Data represent the mean ± SEM of expression values for 4 to 5 mice per group from 2 experiments. (**D**) Mixed WT/*Ifitm3^–/–^* bone marrow chimeras were generated and infected with MCMV. After 4 days, IL-6 in spleen supernatants was quantified by ELISA. Data from 1 of 2 experiments are shown. (**E**) IFITM3 expression by splenic macrophages (Macs), cDCs, and pDCs was assessed by flow cytometry (blue line = WT on day 0, green line = WT on day 2 p.i., red line = *Ifitm3^–/–^* on day 2 p.i.). (**F**) WT and *Ifitm3^–/–^* FLT3L-, GM-CSF– and M-CSF–generated myeloid cells and primary MEFs were infected with MCMV (MOI = 1), and IL-6 protein was measured 6 hours later. Data represent the mean ± SEM of 4 quadruplet wells for at least 3 experiments. *P < 0.05, **P < 0.01, and ***P < 0.001, by 2-tailed Students *t* test (**A**–**C**, **F**) and by 1-way ANOVA with Bonferonni’s multiple comparison post-test analysis (**D**).

Overexuberant cytokine production is associated with virus-induced lymphopenia (30) and with reduced NK cell accumulation during acute MCMV infection (34). We therefore hypothesized that unregulated cytokine production was driving the loss of cellular antiviral immunity in this model. Small but significant elevations of IL-12p70, GM-CSF, and IFN-α were detected in splenic homogenates from *Ifitm3^–/–^* mice (Figure 4A). We also observed a moderate increase in TNF-α expression by day 4 p.i. (Figure 4A), a result reproduced in 3 of 4 experiments (data not shown). Importantly, however, dramatic increases in IL-6 protein concentrations were routinely observed in *Ifitm3^–/–^* mice at multiple time points following acute infection (Figure 4, A and B).

IL-6 is implicated in numerous inflammatory pathologies (reviewed in ref. 36). Therefore, we investigated the role that IL-6 played in MCMV-associated pathogenesis. Myeloid cells (conventional DCs [cDCs], macrophages, and plasmacytoid DCs [pDCs]) were identified as primary sources of IL-6 expression during MCMV infection in vivo, and the percentage of myeloid cells expressing IL-6 within these cell populations was substantially elevated in *Ifitm3^–/–^* mice (Figure 4C). Importantly, MCMV infection of chimeric mice expressing IFITM3 in hematopoietic and/or nonhematopoietic cells revealed that hematopoietic cell expression of IFITM3 was essential for controlled IL-6 production (Figure 4D). Macrophages, cDCs, and pDCs all expressed IFITM3 during MCMV infection (Figure 4E). We therefore hypothesized that IFITM3 directly inhibited virus-induced IL-6 production by myeloid cells. To test this, we generated myeloid cell cultures from WT and *Ifitm3^–/–^* bone marrow stem cells. Although IFITM3 deficiency did not have a notable impact on the low levels of MCMV-induced IL-6 production by M-CSF–differentiated cells or by MEFs, both GM-CSF– and FLT3L-differentiated *Ifitm3^–/–^* cells produced significantly more IL-6 than did WT cells in response to MCMV (Figure 4F). Importantly, these data were derived from the same experiments in which we confirmed that IFITM3 did not restrict MCMV entry (Figure 2).

**Figure 5:**
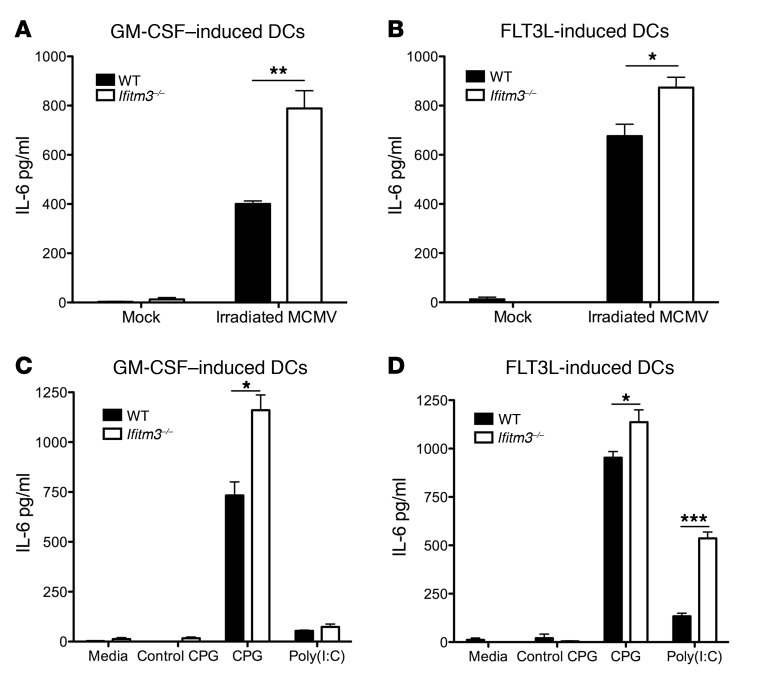
*Ifitm3^–/–^* myeloid cells are hyperresponsive to replication-deficient virus and endosomal TLR ligand stimulation. (**A** and **B**) GM-CSF– and FLT3L-differentiated myeloid cells were infected or not with irradiated MCMV, and IL-6 protein in the supernatants was analyzed by ELISA 24 hours later. (**C** and **D**) GM-CSF– and FLT3L-differentiated myeloid cells were stimulated or not with a control CPG or CPG (both 0.5 μg/ml) or with Poly(I:C) (10 μg/ml) for 24 hours, and IL-6 protein in the supernatants was analyzed by ELISA. Data represent the mean ± SEM of quadruplet wells for 2 (**C** and **D**) or 3 (**A** and **B**) experiments. *P < 0.05, **P < 0.01, and ***P < 0.001, by 2-tailed Students *t* test.

To further confirm that increased MCMV-induced IL-6 production in *Ifitm3^–/–^* myeloid cells was not a consequence of enhanced viral replication and/or cell entry, we incubated GM-CSF– and FLT3L-differentiated myeloid cells with irradiated MCMV and detected increased IL-6 production by *Ifitm3^–/–^* cells (Figure 5, A and B). TLR3 and TLR9 are stimulated by MCMV (37, 38), and we found that *Ifitm3^–/–^* myeloid cells also produced more IL-6 in response to the TLR9 ligand CpG and, in the case of FLT3L-generated cells, the TLR3 ligand Poly(I:C) (Figure 5, C and D). In contrast, IL-6 induction in response to cell-surface–expressed TLR4 and the cytoplasmic DNA sensor stimulator of IFN genes (STING) was not significantly altered by IFITM3 deficiency, although we observed a trend toward increased IL-6 production following stimulation of both innate immune sensors (Supplemental Figure 3). Thus, IFITM3 significantly suppresses myeloid cell regulation of IL-6 production in response to endosomal TLR ligands and replicating and nonreplicating MCMV.

### IL-6R signaling mediates MCMV-induced pathogenesis.

**Figure 6:**
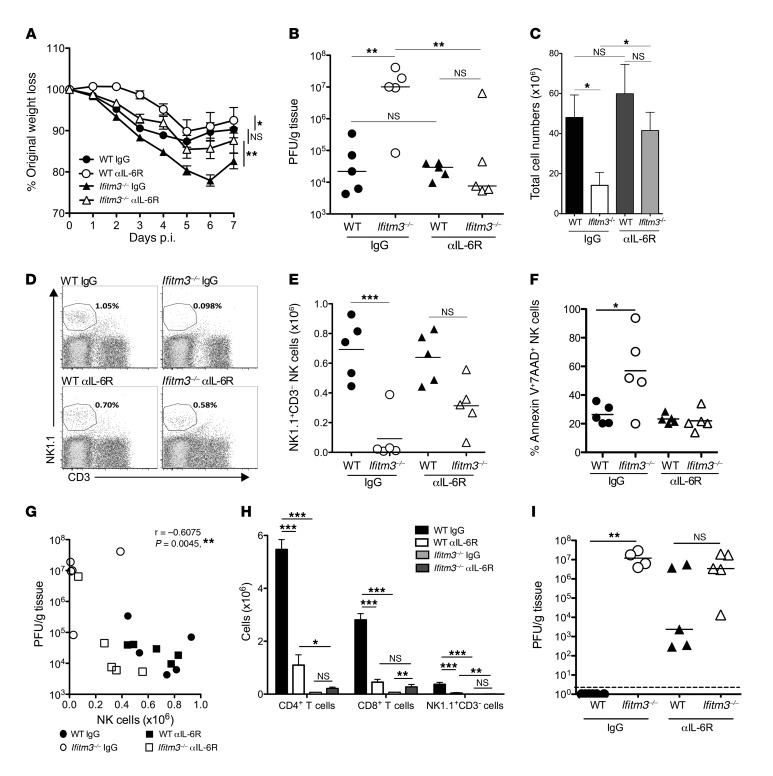
IL-6 is a critical regulator of MCMV-induced pathology in *Ifitm3^–/–^* mice. WT and *Ifitm3^–/–^* mice were infected with 3 × 10^4^ PFU MCMV and treated with IgG or anti–IL-6R (2B10) on days 0 and 4 p.i. (**A**) Weight loss was measured over a 7-day period. Data represent the mean ± SEM of 4 to 11 mice per group. (**B**) Viral load in the spleen was quantified by plaque assay 4 days p.i. Data represent individual mice ± the median for 2 experiments. (**C**) Viable splenocytes were counted on day 4 p.i. Data represent the mean ± SEM of 2 merged experiments using 9–11 mice per group. (**D**) Representative bivariate plots of NK1.1 versus CD3 expression in WT (left) and *Ifitm3^–/–^* (right) mice 4 days p.i. after IgG (top) or anti–IL-6R (αIL-6R) (bottom) treatment. Viable NK cells (**E**) and annexin V^+^7AAD^+^ NK cells (**F**) were quantified 4 days p.i. (**G**) Correlation between viral load and NK1.1^+^CD3^–^ cells in chimeric WT/*Ifitm3^–/–^* mice treated with IgG or anti–IL-6R. (**H** and **I**) After 7 days, viable splenic T cells and NK1.1^+^ cells were quantified and expressed as the mean ± SEM for 4 to 6 mice per group (**H**), and viral load in the spleen was measured (**I**). All results represent 2–3 experiments. *P < 0.05, **P < 0.01, and ***P < 0.001, by 1-way ANOVA (**A**); 1-way ANOVA with Bonferonni’s multiple comparison post-test analysis (**B**); 2-tailed Students *t* test (**C**, **E**, **F**); Mann Whitney-U (**I**); 1-way ANOVA (**H**); Spearman’s rank (**G**).

We assessed whether enhanced cytokine production was responsible for the pathogenesis observed in *Ifitm3^–/–^* mice. Administration of an antagonist anti–IL-6 receptor (anti–IL-6R) monoclonal antibody that blocks both classical IL-6R signaling and IL-6 trans-signaling in vivo (39) significantly alleviated virus-induced weight loss in WT and *Ifitm3^–/–^* mice (Figure 6A). Here, IL-6R blockade halved the loss of weight in *Ifitm3^–/–^* mice seen during the first 4 days of infection (Figure 6A). In contrast, neutralization of TNF-α had no impact on MCMV-driven weight loss in *Ifitm3^–/–^* mice (Supplemental Figure 4). These data suggested a dominant role for IFITM3 regulation of IL-6 production in determining the pathological outcome of MCMV infection.

The improved outcome of acute infection after anti–IL-6R treatment of *Ifitm3^–/–^* mice was accompanied by a dramatic reduction in viral replication (4 days p.i.). The viral load in these antibody-treated animals resembled the levels observed in infected WT mice (Figure 6B). This was associated with a recovery of leukocyte (Figure 6C) and NK cell accumulation at this time (Figure 6, D and E). Unregulated cytokine production during acute MCMV infection promotes NK cell death (34). Interestingly, IL-6R blockade completely reversed the accumulation of annexin V^+^7AAD^+^ NK cells (Figure 6F). Correlation analysis of NK cell accumulation versus PFU of all treated and untreated WT and *Ifitm3^–/–^* mice revealed a significant inverse correlation between NK cell numbers and virus control (Figure 6G), whereas viral load and CD3^+^ T cell accumulation showed no statistically significant correlation (Supplemental Figure 5A). Thus, inhibition of IL-6R signaling alleviates early virus-induced disease in *Ifitm3^–/–^* hosts. These findings support a role for IL-6 in the control of viral replication and NK cell survival.

### IL-6R signaling is required for the development of antiviral cellular immunity.

The beneficial impact of anti–IL-6R treatment was reduced at later time points of infection in both WT and *Ifitm3^–/–^* mice (Figure 6A). IL-6R blockade failed to reverse the dramatic loss of T cell accumulation in the spleens of *Ifitm3^–/–^* mice 7 days p.i. (Figure 6H), without selectively antagonizing the accumulation of particular lymphocyte subsets (Supplemental Figure 5B). The failure of IL-6R blockade to rescue T cell responses did not reflect a cell-intrinsic role for IFITM3 in maintaining T cell responses in vivo, as demonstrated by the comparable recovery of WT and *Ifitm3^–/–^* CD4^+^ and CD8^+^ T cells following MCMV infection (Supplemental Figure 5C). Instead, in accordance with data derived from influenza infection models (40), IL-6 was required for the development of virus-specific T cell responses in MCMV (Supplemental Figure 5D). Furthermore, NK cell accumulation at this time was also dependent on IL-6, and anti–IL-6R treatment also failed to rescue the loss of NK cells in *Ifitm3^–/–^* mice (Figures 6H and Supplemental Figure 5B). Consequently, the early control of viral replication observed following IL-6R blockade in *Ifitm3^–/–^* mice was not sustained at 7 days p.i. (Figure 6I). These data highlight IL-6 as an important mediator of viral pathogenesis and suggest a critical role for IFITM3 in the appropriate temporal regulation of the production of this cytokine in response to herpesvirus infection.

## Discussion

Experimental evidence to date has suggested that exacerbated viral pathogenesis in hosts with deficient or impaired IFITM3 activity is a consequence of impaired restriction of virus entry and subsequent replication. Here, we provide evidence that direct regulation of virus-induced cytokine production is an important additional in vivo function of IFITM3 and, in the context of CMV infection, represents the dominant mechanism through which IFITM3 controls virus-induced disease.

We demonstrated that the production of multiple cytokines were restricted by IFITM3. However, our data define IFITM3 regulation of IL-6 production as a central determinant of the pathogenesis associated with MCMV infection. In the early stages of infection, IL-6 was the key driver of virus-induced weight loss in WT and *Ifitm3^–/–^* mice and impinged on cellular antiviral immunity. These findings are consistent with observations that IL-6 drives lymphopenia (41), inhibits lymphopoiesis (42), and induces glucocorticoid expression (43). Further, our data suggest that IL-6–induced, apoptosis-independent lymphocyte death was associated with lymphopenia in MCMV-infected *Ifitm3^–/–^* mice. Although it is not possible to discriminate directly ex vivo among different forms of apoptosis-independent cell death, IL-6 may promote cell death directly via the induction of autophagy (44) or STAT3-mediated necrosis (45). Alternatively, IL-6 may indirectly trigger cell death via the induction of multiple cytokines implicated in the activation-induced death of T cells and NK cells (46, 47). Given that IL-6 was also required for the latter accumulation of NK cells and virus-specific T cells during MCMV infection, IL-6 likely exerts differential context-dependent pro-death or pro-survival signals in leukocytes during infection (reviewed in ref. 36).

NK cells exert critical control of HCMV (32) and MCMV (2) infections. Although we cannot formally exclude the possibility that IFITM3 may influence viral replication in an unidentified cell type in vivo, the observation that viral load after 4 days of infection was comparable in WT and *Ifitm3^–/–^* mice following NK cell depletion or after challenge with Δm157 MCMV strongly suggests that impaired NK cell responses were primarily responsible for the elevated viral replication in *Ifitm3^–/–^* mice. In this regard, IL-6R blockade was also found to rescue both NK cell responses and viral control in *Ifitm3*^–/––^ mice. Interestingly, however, although IL-6 has recently been implicated in the direct control of HCMV replication (48), inhibiting the action of IL-6 restricts HCMV reactivation from latency in DCs (49). Thus, it is possible that the beneficial impact of anti–IL-6R treatment on the control of viral replication in our experiments may extend beyond the restriction of lymphopenia and NK cell death.

IFITM3 restricted myeloid cell production of IL-6 upon exposure to MCMV in vitro. IFITM3 expression by hematopoietic cells was critical for the controlled production of IL-6 in vivo, and the frequency of IL-6–secreting myeloid cells was elevated in MCMV-infected *Ifitm3^–/–^* mice. The latter observation may, in part, reflect a feedback mechanism through which elevated viral load in *Ifitm3^–/–^* mice further stimulated myeloid cell production of IL-6 and hence increased the frequency of myeloid cells capable of producing IL-6 ex vivo. Of critical importance, however, we demonstrate that *Ifitm3^–/–^* myeloid cells produce more IL-6 in response to nonreplicating virus, and IL-6R blockade is sufficient to restore control of MCMV replication in *Ifitm3^–/–^* mice. Thus, IFITM3 acts as an immune regulator that restricts virus-induced IL-6 production by myeloid cells independently of controlling virus entry and replication. The IFITM3/IL-6 axis is therefore responsible for determining viral pathogenesis in vivo.

TLRs located in endosomes are triggered by MCMV (37, 38) and induce the expression of cytokines including IL-6 upon stimulation (50). Of critical importance, our data reveal that IFITM3 suppresses TLR3- and TLR9-induced cytokine production by myeloid cells in response to nonreplicating ligands. In contrast, IL-6 production by *Ifitm3^–/–^* cells was only moderately elevated following stimulation of cell membrane–expressed TLR4 or the cytoplasmic DNA sensor STING. These data suggest that IFITM3 may preferentially regulate the activation and/or downstream signaling triggered by endosomal TLRs and that this represents a broad regulatory function not restricted to antiviral responses. IFITM3 localization within endosomes may be critical for its regulatory function either directly or by influencing the entry of virus, TLR ligands, or TLRs into the endosomal pathway. Importantly, however, a trend of increased cytokine expression in response to STING and TLR4 ligands also suggests that the regulatory function(s) of IFITM3 extend beyond the modulation of endosomal TLR activation. A role for IFITM3 in membrane trafficking has been reported (51), and thus IFITM3 may influence downstream events induced by innate pathogen recognition such as expression or secretion of cytokines and/or cytokine receptor activity. Understanding the exact function of IFITM3 within virus-exposed, virus-infected, and uninfected cells will be an important future area of study.

The overt cytokine production observed in MCMV-infected *Ifitm3^–/–^* mice had some similarities to the inflammatory disease hemophagocytic lymphohistiocytosis (HLH). Herpesvirus infections, particularly EBV, are common triggers of HLH, and EBV-associated HLH is highly prevalent in Asia, suggesting an influence of host genetics on the disease (52). Our data imply that genetic variation in genes encoding proteins such as IFITM3 that exhibit immune modulatory functions may influence the occurrence and/or severity of herpesvirus-triggered HLH. The data derived from our in vivo model also imply that individuals with the minor IFITM3 rs12252-C SNP may also have altered susceptibility to HCMV disease.

An important implication of our data is that individuals with reduced IFITM3 activity suffer from virus-induced pathogenesis that is driven, at least part, by unregulated cytokine production. In support of this hypothesis, heightened early production of inflammation-associated cytokines including IL-6 is associated with the fatal outcome of influenza H7N9 infection (53), consistent with a role for inflammation in driving influenza-associated diseases (54–56). Of critical importance, Wang et al. demonstrated the rs12252-C SNP to be a concurrent risk factor of fatal influenza infection (53). We now provide what we believe to be direct evidence of a link between excessive cytokine production as a consequence of impaired IFITM3 function and fatal viral infection.

Overall, our data demonstrate that restriction of virus-induced cytokine production is an important and previously unexplored mechanism through which IFITM3 regulates both virus-induced pathogenesis and that this process exerts a critical influence on the outcome of CMV infection. Although the long-term benefits of IL-6R blockade were limited due to the latter requirement of this cytokine pathway in cellular immune responses including the development of virus-specific T cell immunity, these results highlight the idea that reducing overt cytokine production, perhaps using more subtle and/or broader approaches, may represent an effective strategy for the treatment of virus-infected individuals with impaired IFITM3 activity. Finally, the discovery that IFITM3 inhibits TLR-mediated cytokine production may provide insight into why the rs12252-C SNP has been conserved in human populations, despite its potential deleterious effect associated with reduced IFITM3 function.

## Methods

### Mice, viral infections, and treatments.

IFITM3-deficient (*Ifitm3^–/–^*) and WT control (95% C57BL/6, 5% 129) mice have been described previously (16). Age- and sex-matched mice between 7 and 12 weeks of age were used in the experiments. The MCMV Smith strain (ATCC) for in vivo experimentation was prepared in the salivary glands of 3- to 4-week-old BALB/c mice. Salivary glands were homogenized and virus from supernatant purified over a sorbital gradient. Virus was passaged no less than 3 and no more than 5 times in vivo. Virus from homogenized organs and tissue culture supernatants was titered for 6 days on 3T3 cells with a carboxymethylcellulose overlay. Mice were infected i.p. with 3 × 10^4^ PFU MCMV. For NK cell depletion, mice were injected i.p. with 200 μg anti-NK1.1 (clone PK136; Bio X Cell) or IgG control on days −2, 0, and +2 p.i., or in repeat experiments, mice were injected i.p. with 250 μg αAsialo-GM1 polyclonal antibody (eBioscience) or PBS control on days –3 and 0 p.i. For IL-6R blockade, mice were injected i.p. with 300 μg anti–IL-6R (clone 2B10 made in-house) or IgG control on day 0, and for 7-day experiments, on day 4 p.i. For WT/*Ifitm3*^–/–^ bone marrow chimeras, mice were irradiated at 2 × 4.5 Gy and transfused i.v. with 1 × 10^6^ bone marrow cells 24 hours later. Mice were then treated for 2 weeks with antibiotic-supplemented water. Mice were infected with MCMV 8 weeks after irradiation.

### Leukocyte isolation, intracellular cytokine staining, and flow cytometry.

Leukocytes (1 × 10^6^) were stained, in most experiments, with Zombie Aqua dye, incubated with Fc block (both from BioLegend), and stained for surface markers with a combination of the following antibodies (all from BioLegend, eBioscience, or BD Biosciences): anti-NK1.1 (clone PK136); anti-CD3ε (clone 145-2C11); anti-CD25 (clone 3C7); anti-CD4 (clone GK1.5); anti-CD8 (clone 53-6.7); anti-CD11b (clone M1/70); anti-CD11c (clone N418); anti-CD45R/B220 (clone RA3-6B2); anti-F4/80 (clone BM8); anti–I-A/I-E (clone M5/114.15.2); anti-Ly6G (clone 1A8); and anti–Siglec H (clone 551). Following surface staining, some cells were fixed and permeabilized with saponin buffer (PBS, 2% FCS, 0.05% sodium azide, and 0.5% saponin) and stained with anti–IL-6 (clone MP5-20F3; BioLegend) or rabbit polyclonal anti-IFITM3 (Abcam), followed by anti-rabbit phycoerythrin (PE) (Sigma-Aldrich). For analysis of NK cell function, cells were incubated for 5 hours in monensin (BD Pharmingen) and anti-CD107a (clone 1D4B; BioLegend), stained with anti-NK1.1 and anti-CD3ε, permeabilized and then stained with anti–IFN-γ (clone XMG1.2; BioLegend). Other unfixed cell preparations were stained with 7AAD and annexin V (both from BioLegend). M45-specific CD8 T cell responses (57) and functional NK cell responses were assessed as described previously (34).

At least 20,000 leukocytes were analyzed using a BD FACSCanto II flow cytometer (BD Biosciences). Electronic compensation was performed with Ab capture beads stained separately with individual mAbs used in the experimental panel. Data were analyzed using FlowJo software, version 8.5.3 (Tree Star). Total numbers of different cell populations were calculated by multiplying the total number of viable leukocytes (assessed by trypan blue exclusion) by the percentage of positive cells, as detected by flow cytometry.

### Peripheral leukocyte, platelet, and red blood cell assessment.

Small (50-μl) blood samples were collected from the lateral tail vein into K2 EDTA-coated 0.1-ml pediatric tubes with an integrated capillary (Kabe Labortechnik GmbH) for determination of complete blood counts using a Scil Vet ABC system.

### In vitro infections.

Bone marrow cells were incubated at 2 × 10^5^ cells/ml or 4 × 10^5^ cells/ml in R10 media supplemented with 20 ng/ml M-CSF (BioLegend) or with 50 μM 2-mercaptoethanol (Gibco, Thermo Fisher Scientific) and 20 ng/ml GM-CSF (BioLegend) for 7 days, respectively. Media was replenished after 3 days. For bone marrow–derived FLT3L-induced myeloid cells, cells were incubated at 4 × 10^5^ cells/ml in R10 media supplemented with 50 μM 2-mercaptoethanol and 100 ng/ml FLT3L (BioLegend) for 9 days, with replenishment of media on days 4 and 8. Myeloid cells were then mock infected or infected with MCMV (using the pSM3fr-MCK-2fl BACmid, a gift of Chris Benedict [La Jolla Institute for Allergy and Immunology, San Diego, USA] and Barbara Adler [Ludwig-Maximilians-University, Munich, Germany]) at an MOI of 1 or 0.1. Some cells were incubated with pSM3fr-MCK-2fl that was irradiated (2,520 Gy) and confirmed as replication deficient by failed infection of 3T3 cells. Some cells were treated with 80 μM EIPA (Sigma-Aldrich) 30 minutes prior to infection, whereas others were treated with 10 μg/ml Poly(I:C) (Sigma-Aldrich) or CpG ODN2395 or ODN2395 control (ODN5328) (Miltenyi Biotec). After 6 or 24 hours, supernatants were collected for IL-6 protein analysis.

To quantify viral infection, M-CSF– and GM-CSF–generated myeloid cells were infected with pSM3fr-MCK-2fl BACmid MCMV (MOI of 1 or 0.1), and 24 hours later cells were stained with Zombie Aqua dye and Fc block, followed by staining for surface markers as described above prior to fixation and permeabilization and intracellular staining with anti-m06 antibody (CapRi) conjugated with allophycocyanin (APC) (Innova Biosciences). Some cells were incubated for 4 days, and the supernatant was analyzed for infectious virus by plaque assay.

To generate primary fibroblasts, adult *Ifitm3^–/–^* mice were intercrossed, and MEFs were derived from embryos on day 13.5 of gestation, as described previously (13). MEFs were cultured in DMEM containing 10% FBS, 1× MEM essential amino acids, and 1× 2-mercaptoethanol (Gibco, Thermo Fisher Scientific). MEFs were then infected with MCMV as described above, and replicating virus in the supernatant was quantified by plaque assay 4 days later.

### Cytokine and chemokine analysis.

IL-6 protein was measured by ELISA (BioLegend). Proinflammatory cytokines were measured using a ProcartaPlex Multiplex Immunoassay Kit (eBioscience) and run on a Bio-Plex 200 Luminex machine (Bio-Rad).

### Histology.

For histological examination, tissues from MCMV-infected tissues organs were fixed in 4% formaldehyde and then processed on paraffin blocks. Five-micrometer sections of paraffin-embedded tissue were stained with H&E (Sigma-Aldrich).

### Statistics.

For viral load analysis, statistical significance was determined using the Mann-Whitney *U* test for comparison of WT and *Ifitm3^–/–^* groups. To analyze viral load data from multiple groups given different treatments, data were first subjected to logarithmic transformation, and subsequent 2-way ANOVA analysis was performed. For paired analysis of flow cytometry data and ELISA data, the 2-tailed Student’s *t* test was used. One-way ANOVA was adopted for analysis of data derived from >2 groups of mice and grouped weight loss data. A *P* value of less than 0.05 was considered statistically significant.

### Study approval.

All animal studies were performed at the Wellcome Trust Sanger Institute (WTSI) research support facility under UK Home Office Project License number 80/2596, as approved by the UK Home Office, London, United Kingdom.

## Author contributions

MAS, SC, MM, JAK, MC, CAF, SES, RSW, SGB, GS, LK, KH, CB, GN, ELC, CI, AOS, and IRH conducted the experiments. MAS, SC, CAF, AOS, ZJ, WF, SAJ, PK, and IRH designed the experiments and analyzed and interpreted the data. ZJ and WF provided key reagents. MAS, SC, SAJ, PK, and IRH wrote the manuscript.

## Supplementary Material

Supplemental data
