# Supplementary material for: The antiviral restriction factor IFN-induced transmembrane protein 3 prevents cytokine-driven CMV pathogenesis
Source: J Clin Invest. 2017 Feb 27;127(4):1463–74. doi: 10.1172/JCI84889 (PMC5373880; doi:10.1172/JCI84889)
Supplement: Supplemental data [file jci-127-84889-s001.pdf]

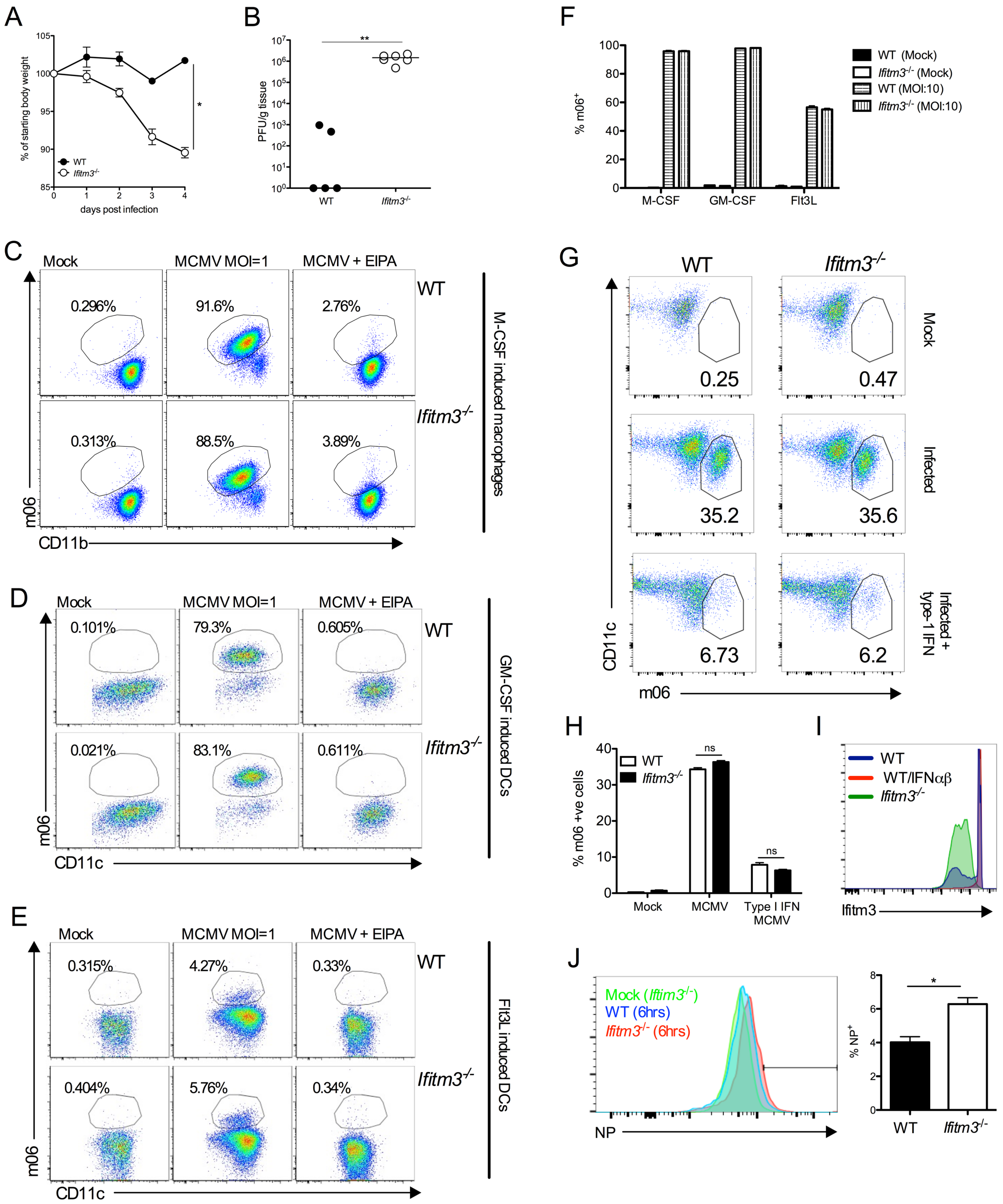

Figure S1. Ifitm3 restricts MCMV pathogenesis but does not directly restrict MCMV replication. (A&B) MCMV-induced weight loss (A) and virus load in spleen 4 days pi (B). Data from 1 of 2 experiments is shown. M-CSF (C), GM-CSF (D) and Flt3L (E) differentiated WT and *Ifitm3*$^{-/-}$ myeloid cells were infected or not with pSM3fr-MCK-2fl MCMV (MOI=1) +/- pre-treatment with the endocytosis inhibitor EIPA. (F) Cells were infected with MOI=10. After 24hrs, virus infection was quantified with staining for intracellular m06. Results are shown as representative FACS plots of at least 3 experiments (C-E) or mean + SEM of biological triplicates (F). (G-I) GM-CSF differentiated bone marrow cells were treated/not with 1000U/ml IFN α and β for 16hrs, and infected with MCMV. After 24hrs, MCMV m06 (G-H) and Ifitm3 (I) expression by CD11c^{+}MHCII^{+} cells were assessed. (H) Data is shown as Mean + SEM of biological triplicates.

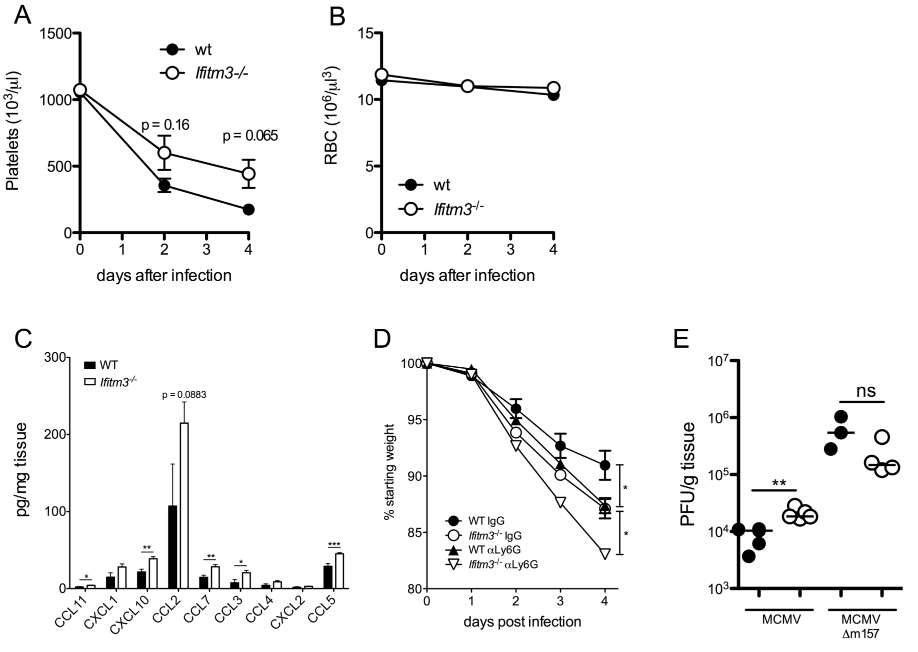

Figure S2. Circulating platelets, red blood cell levels, tissue chemokine production, and the role of neutrophils and NK cell responses in *Ifitm3*$^{-/-}$ mice. Platelet (A) and red blood cell (B) levels were quantified over a 4-day time-course of MCMV infection in WT and *Ifitm3*$^{-/-}$ mice. Mean +/- SEM of 6-9 mice/ group is shown. (C) Chemokine protein in spleen homogenates of WT and *Ifitm3*$^{-/-}$ mice was measured 4 days pi. Mean + SEM of 8-9 mice/group is shown. (D) Weight loss in WT and *Ifitm3*$^{-/-}$ mice +/- neutrophil depletion. Mean +/- SEM of 3-6 mice is shown. (E) Virus titers in spleens 4 days after infection with K181 or delta-m157 MCMV. Individual mice and medians are shown.

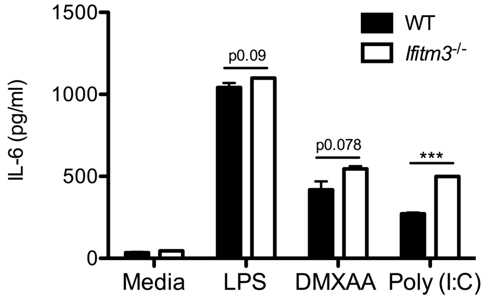

Figure S3. IL-6 production by *Ifitm3*$^{-/-}$ DCs following TLR4 and STING activation. WT and *Ifitm3*$^{-/-}$ DCs were stimulated with 1μg/ml LPS or 100μg/ml DMXAA, and IL-6 production was measured by ELISA after 24hrs. Mean + SEM of biological triplicates in shown and one of two experiment is shown.

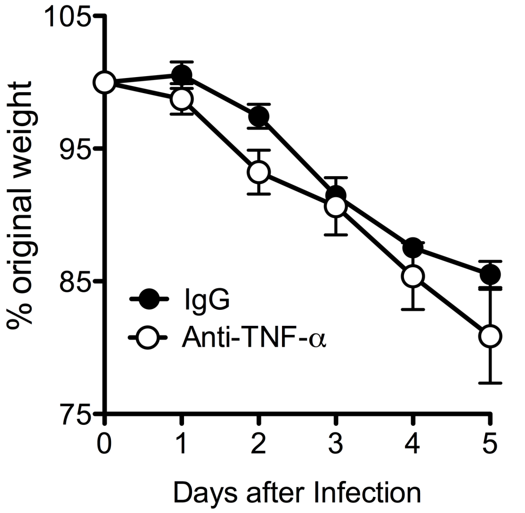

Figure S4. TNF-α neutralization does not impact on MCMV-induced weight loss in *Ifitm3$^{-/-}$* mice. *Ifitm3$^{-/-}$* mice (n=5/group) were infected with MCMV and treated with anti-TNF-α or isotype control, and weight loss was assessed. Mean +/- SEM of % original weight is shown. 1 of 2 experiments is depicted.

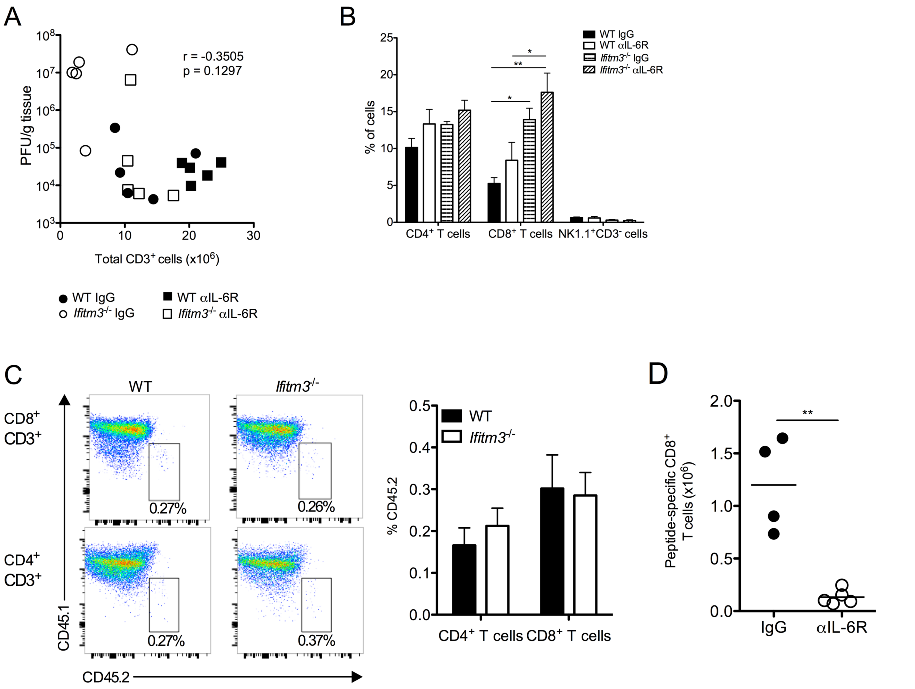

Figure S5. IL-6R is required for the generation of virus-specific T cell responses. (A) WT and *Ifitm3*$^{-/-}$ mice were treated with IgG or anti-IL-6R. After 4 days the correlation between CD3$^+$ cells and virus PFU in the spleen was assessed. (B) % values of splenic CD4$^+$, CD8$^+$ and NK1.1$^+$ cells from WT and *Ifitm3*$^{-/-}$ mice 7 days pi following treatment with IgG or anti-IL-6R. Mean + SEM from 4-5 mice/group is shown from 1 of 2 experiments. (C) Accumulation of CD45.2 WT and *Ifitm3*$^{-/-}$ CD4$^+$ and CD8$^+$ T cells 7 days after infection of recipient CD45.1 mice. Data is shown as representative bivariant FACS plots (left) or mean + SEM of 6 (WT) or 4 (*Ifitm3*$^{-/-}$) mice/group. (D) WT mice were treated with IgG or anti-IL-6R and splenic virus-specific CD8$^+$ T cells were assessed by IFN-γ detection after stimulation with peptide. Individual mice + mean is shown from 1 of 2 experiments.
